# Rat Extermination

**Published:** 1908-01-18

**Authors:** 


### RAT EXTERMINATION.

As the result of a meeting held at the Hôtel Métropole on January 10, under the chairmanship of Sir James Crichton-Browne, a national society has been formed to

"... . determine
What's best to rid us of our vermin."

Throughout the Chairman's indictment of the rat one was forcibly reminded of the second stanza of Robert Browning's delightful poem, "The Pied Piper of Hamelin." Sir James Crichton-Browne summed up in a most convincing manner the widespread evils which are known nowadays to be due to this repulsive and prolific animal. The worthy citizens of Hamelin town were only too well aware of the destructive effects of rats upon property. Had they suspected that these pests were also guilty of the dissemination of disease among human beings it is probable that the Mayor and Corporation would have paid the Piper his promised thousand guilders without demur at the conclusion of his task.

Sir James Crichton-Browne, in his speech for the prosecution, referred to the connection, established by Zuschlag, between rats and trichinosis, and then passed to a far more serious side of the matter, the propagation of plague by rats. We in England are fortunately not at present menaced by bubonic plague; but the death-rate from this disease among our fellow subjects in India is appalling—since 1896 no less than five and a quarter million deaths have been due to this cause. Rat destruction is, therefore, a matter of the highest importance in the Indian Empire, but, at the same time, there can be no doubt that the problem presents many and great difficulties. Some authorities even go so far as to say that rat extermination in that country is an impossibility, and that other methods for the prevention of plague must be discovered. It is to be hoped that this view will prove to be too pessimistic.

Although in England there is not the same urgent necessity, on the grounds of public health, for measures to be taken, yet there is already ample excuse for an organised wholesale slaughter of vermin in this country. The headquarters of such an imperial crusade would quite appropriately be situated in the mother country, and we welcome with all cordiality the first stage of this important undertaking.

Sir Lauder Brunton was elected president of the new Society, and he is supported by several other prominent members of the medical profession, by scientists, and by agriculturists. The treasurer is Lord Avebury, and the secretary Mr. A. E. Moore. As soon as the committee has evolved and organised its scheme for rat extermination it will no doubt take the public into its confidence. In the meantime it may be gathered from the Chairman's opening speech that the Society will not confine itself to efforts in any one direction, but will include in its plan of attack those methods which have already been tried and found of service. Dogs, cats, ferrets, guns, traps, and poison have all been used in the past, some of them separately, others in combination. Now we have modern scientific methods whose common aim is to inoculate rats with some specific disease which runs an epidemic course among rats, while leaving other animals unaffected. The "Danysz Virus," invented by Dr. Danysz of the Pasteur Institute, is one of the best known of such methods. Another substance which has lately formed the subject of satisfactory practical trials by the Somersetshire Chamber of Agriculture is that known as "Ratin." Whatever may be the means adopted by the Society, we may be sure that they will first be the subject of careful thought and inquiry; and the Committee can count upon the willing co-operation of the medical profession and of the British public.

